# PPARβ/δ selectively regulates phenotypic features of age-related macular degeneration

**DOI:** 10.18632/aging.101031

**Published:** 2016-09-08

**Authors:** Mayur Choudhary, Jin-dong Ding, Xiaoping Qi, Michael E. Boulton, Pei-Li Yao, Jeffrey M. Peters, Goldis Malek

**Affiliations:** ^1^ Department of Ophthalmology, Duke University School of Medicine, Durham, NC 27703, USA; ^2^ Department of Ophthalmology, Indiana University School of Medicine, Indianapolis, IN 46202, USA; ^3^ Department of Veterinary and Biomedical Sciences, The Pennsylvania State University, University Park, PA 16802, USA; ^4^ Department of Pathology, Duke University School of Medicine, Durham, NC 27703, USA

**Keywords:** age-related macular degeneration, PPARβ/δ, nuclear receptors, inflammation, angiogenesis, choroidal neovascularization

## Abstract

Peroxisome proliferator-activated receptor-β/δ (PPARβ/δ) is a nuclear receptor that regulates differentiation, inflammation, lipid metabolism, extracellular matrix remodeling, and angiogenesis in multiple tissues. These pathways are also central to the pathogenesis of age-related macular degeneration (AMD), the leading cause of vision loss globally. With the goal of identifying signaling pathways that may be important in the development of AMD, we investigated the impact of PPARβ/δ activation on ocular tissues affected in the disease. PPARβ/δ is expressed and can be activated in AMD vulnerable cells, including retinal pigment epithelial (RPE) and choroidal endothelial cells. Further, PPARβ/δ knockdown modulates AMD-related pathways selectively. Specifically, genetic ablation of *Pparβ/δ* in aged mice resulted in exacerbation of several phenotypic features of early dry AMD, but attenuation of experimentally induced choroidal neovascular (CNV) lesions. Antagonizing PPARβ/δ in both *in vitro* angiogenesis assays and in the *in vivo* experimentally induced CNV model, inhibited angiogenesis and angiogenic pathways, while ligand activation of PPARβ/δ, *in vitro*, decreased RPE lipid accumulation, characteristic of dry AMD. This study demonstrates for the first time, selective regulation of a nuclear receptor in the eye and establishes that selective targeting of PPARβ/δ may be a suitable strategy for treatment of different clinical sub-types of AMD.

## INTRODUCTION

Age-related macular degeneration (AMD) is the leading cause of vision loss in the elderly in the Western World. It is a complex disease as reflected not only by the ever growing number of genetic, environmental, and systemic risk factors identified to date [1-3], but also our improved understanding of the various clinical phenotypes, through the advent of high resolution imaging modalities used to evaluate both patients and post-mortem tissue pathology [4-8]. Clinically, during the initial stages of AMD development, known as ‘early dry’, patients accumulate extracellular lipid and protein filled deposits below the retinal pigment epithelial (RPE) cell layer [1, 5, 9], which normally serves as crucial support to the overlying neural retina and forms part of the outer blood retina barrier [10]. These deposits can lead to dysfunction and atrophy of RPE cells, which along with loss of photoreceptors and choroidal endothelial cells, are major steps in AMD progression towards severe vision deterioration associated with the ‘late dry’ sub-type known as geographic atrophy [1, 3, 11]. Advanced exudative or ‘wet’ AMD is characterized by endothelial invasion through Bruch's membrane and pathological growth of abnormal new vessels originating from the choroid, below the RPE, resulting in the formation of choroidal neovascular (CNV) lesions, which are responsible for the most severe form of disease-related vision loss [1, 3, 12]. Demographically, both the increase in life expectancy of the general population, and prevalence of AMD in the elderly population with age [2, 3], ensures that this disease will become an even greater health problem in the near future. Therefore, it is imperative to investigate the pathological pathways that are altered in this complex disease, recognize potential models that demonstrate phenotypic features of AMD, and identify alternative targets, in order to develop treatments and improve the quality of life of patients. Currently there are no therapies available for the ‘dry’ forms of the disease. However, antibody-based treatments targeting vascular endothelial growth factor (VEGF) are offered to patients with ‘wet’ AMD, which are effective to varying degrees in approximately 30-60% of the patients. This leaves more than 30% of the patient population, for which an alternative treatment must be found.

Peroxisome proliferator-activated receptors (PPARs) are ligand activated transcription factors, which belong to the steroid hormone superfamily. Though PPARα, PPARβ/δ and PPARγ are ubiquitously expressed throughout the body [13, 14], PPARβ/δ has been shown to be involved in regulation of pathways important in AMD pathogenesis, including lipid metabolism, extracellular matrix remodeling, angiogenesis and inflammation [1, 15]. Because of this, we hypothesized that PPARβ/δ is critical in the etiology of AMD. Here we report a novel role of the PPARβ/δ pathway in the pathobiology of AMD. We first established the expression and activity of the pathway in cell culture models of RPE and choroidal endothelial cells, cells vulnerable in AMD. Next we evaluated the ocular phenotype of aged mice harboring the null allele at the *PPARβ/δ* locus (*Pparβ/δ^−/−^)*. Finally, we tested the therapeutic potential of targeting the PPARβ/δ pathway in an experimentally induced model of choroidal neovascularization. Our *in vitro* studies revealed that following *PPARβ/δ* knockdown there is an increase in expression of select extracellular matrix molecules concomitant with a decrease in expression of growth factors, in both RPE and choroidal endothelial cells. Similarly, *PPARβ/δ* knockdown impacted the expression of several AMD-related genes in the inflammatory and lipid metabolic pathways. *In vivo* evaluation of eyes from aged wild-type mice showed accumulation of thin patchy sub-RPE deposits, while genetic ablation of *Pparβ/δ in vivo,* resulted in increased frequency and severity of continuous sub-RPE deposits along with development of RPE degenerative changes. On the other hand, *Pparβ/δ* knockout mice develop CNV lesions smaller in volume and area, increased localization of immune cells, and decreased deposition of extracellular matrix molecules, compared to *Pparβ/δ*^+/+^ mice. Finally, we observed that treatment with a PPARβ/δ antagonist, GSK0660, resulted in a significant inhibition of neovascular lesion size, and extracellular matrix deposition, in aged mice, while treatment with a PPARβ/δ agonist resulted in a decrease of lipid accumulation in a cell culture model of ‘lipid loaded RPE’ cells. This study establishes a strong basis to consider selectively testing and developing, PPARβ/δ ligands as potential therapies for AMD.

## RESULTS

### The PPARβ/δ pathway is biologically active in AMD vulnerable cells

**Figure 1:**
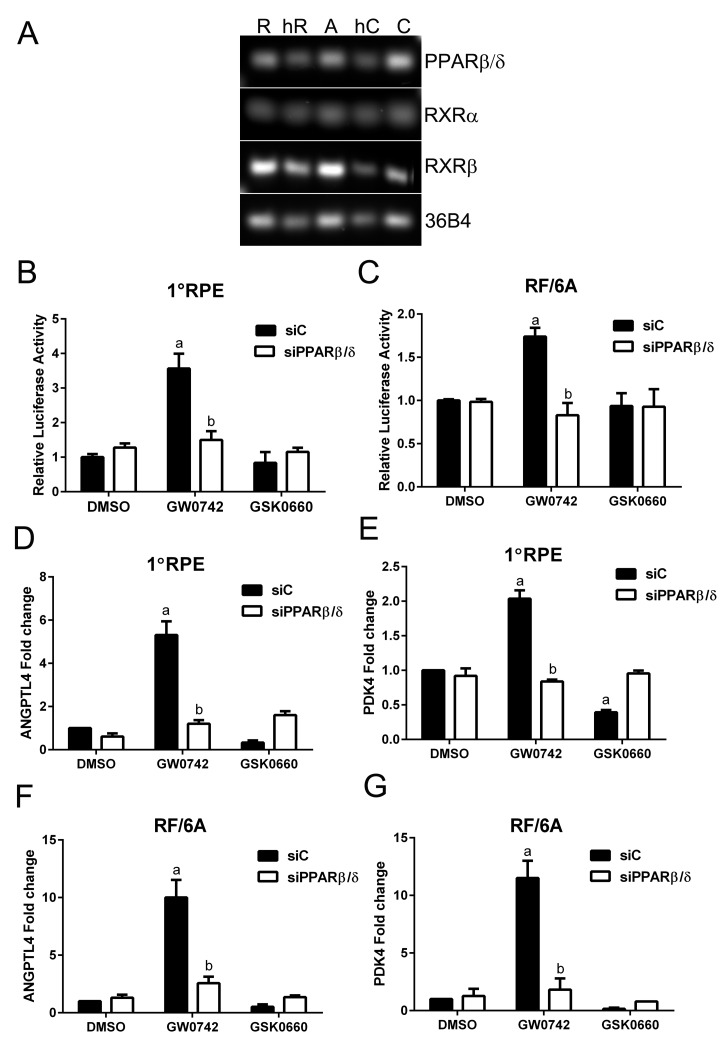
PPARβ/δ signaling pathway is functional in AMD vulnerable cells (**A**) Agarose gel image of PCR amplification products of PPARβ/δ and its obligate binding partners RXRα and RXRβ in primary human RPE cells [R], freshly isolated human RPE cells [hR], ARPE19 cells [A], human choroid [hC], and RF/6A cells [C], 36B4 was used as loading control. PPARβ/δ activity in primary RPE (1°RPE) cells (**B**) and RF/6A cells (**C**) transfected with the DR1 luciferase reporter and siC or siPPARβ/δ; cells were treated with PPARβ/δ agonist, GW0742 (10μM) or antagonist, GSK0660 (10μM) or DMSO as vehicle control (*n =* 3): a: *p <* 0.05 relative to DMSO treated cells; b: *p <* 0.05 relative to drug+siC treated cells (*p* <  0.05; two way ANOVA, Sidak's multiple comparisons test). Expression of *ANGPTL4* and *PDK4* mRNA in primary RPE (1°RPE) cells (**D** and **E**) and RF/6A (**F** and **G**) in siC and siPPARβ/δ (100 pmoles/250,000 cells) treated cells in response to GW0742, GSK0660, or DMSO as a control (*n =* 3); a: *p <* 0.05 relative to DMSO treated cells; b: *p <* 0.05 relative to drug+siC treated cells (Two way ANOVA, Sidak's multiple comparisons test).

The activity of PPARβ/δ in AMD vulnerable cells was examined in human primary RPE cells, the human derived ARPE19, and the macaque derived RF/6A choroidal endothelial cell lines. This was performed by examination of the (i) expression of PPARβ/δ and its binding partners the retinoid X receptor alpha and beta (RXRα and β), (ii) receptor transcriptional activity, by measuring the binding of the receptor-ligand complex to the gene-response element, and (iii) expression of known PPARβ/δ target genes. Freshly isolated human RPE cells and choroid from aged donor eyes, along with primary human RPE cultured cells, ARPE19 and RF/6A cell lines, expressed *PPARβ/δ* and its binding partners the *RXRs* (Figure 1A). Additionally, ligand activation of PPARβ/δ with GW0742 (10μM) caused increased transcriptional activity in human primary RPE (Figure 1B), RF/6A cells (Figure 1C) and ARPE19 cells (Figure SF1) [16]. These changes were mitigated by the PPARβ/δ antagonist, GSK0660 (10μM), and siRNA-mediated knockdown of PPARβ/δ (Figure 1, and C). Similarly, ligand activation of PPARβ/δ increased expression of the PPARβ/δ target genes, angiopoietin-like 4 (*ANGPTL4*) and pyruvate dehydrogenase kinase, isozyme 4 (*PDK4*), in primary RPE cells (Figure 1, and E), RF/6A cells and ARPE19 cells (Figure 1, F, G, S1 and S2), an effect that was diminished by treatment with a PPARβ/δ antagonist or *PPARβ/δ* knockdown.

### Loss of PPARβ/δ results in selective regulation of dry and wet AMD related pathogenic pathways

The discovery of multiple genetic, systemic, and environmental risk factors associated with AMD ontology has resulted in the identification of several AMD-pathogenic pathways. These pathways include, but are not limited to, impairment of extracellular matrix turnover [3, 17], increased angiogenesis [18], inflammation [19, 20], and dysregulation of lipid processing pathways [21]. Since modulation of the PPARβ/δ pathway has been shown to regulate collagen synthesis *in vitro* and *in vivo* [22-24], the effect of *PPARβ/δ* knockdown (*siPPARβ/δ*) on the expression of extracellular matrix-related genes was assessed.

**Figure 2:**
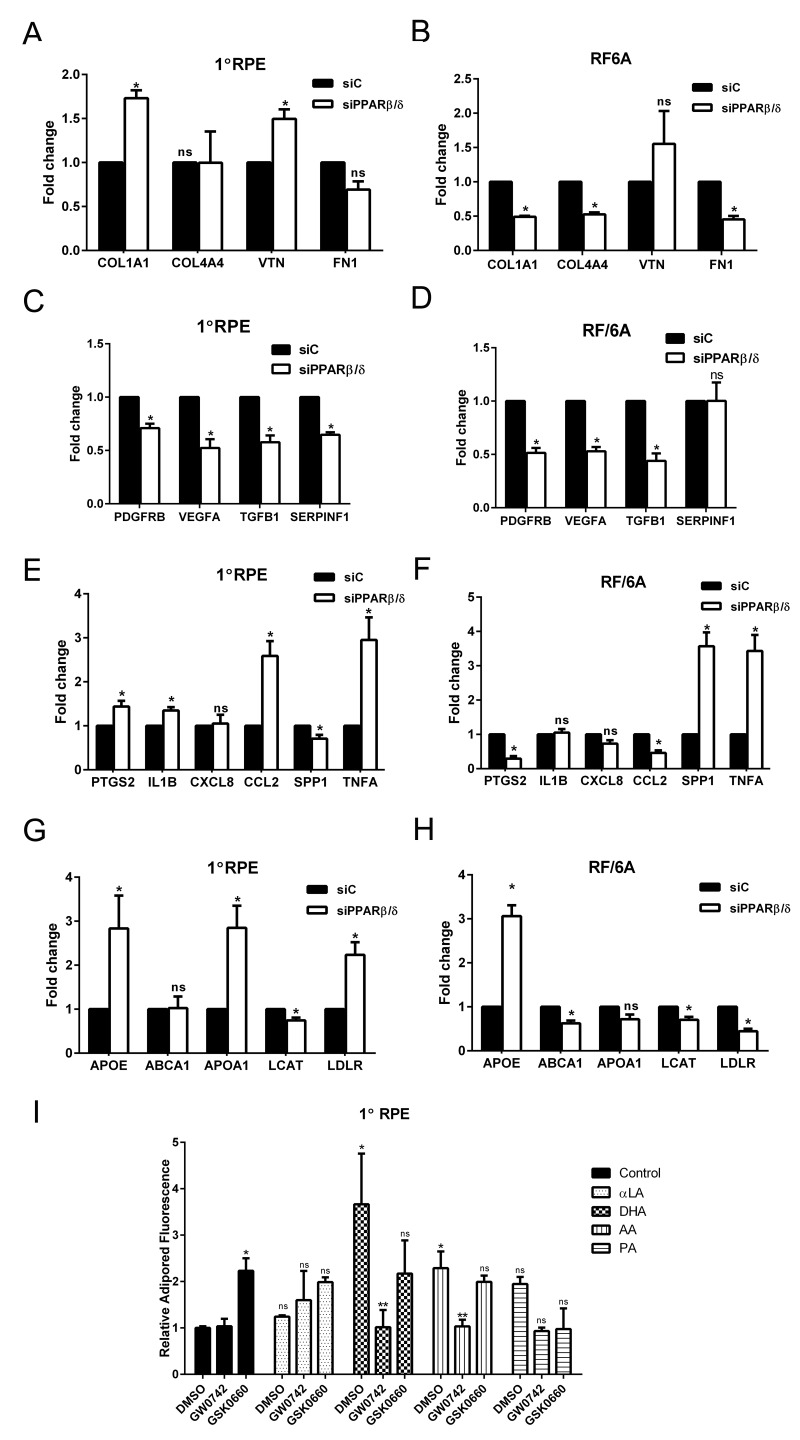
PPARβ/δ regulates both dry- and wet-AMD related pathogenic pathways Effect of siRNA mediated knockdown of *PPARβ/δ* on mRNA expression of extracellular matrix genes COL1A1, COL4A4, FN1 and VTN; angiogenesis and fibrosis genes, *VEGFA*, *PDGFB*, *TGFB1* and *SERPINF1*; inflammation-related genes, *PTGS2*, *IL1B*, *CXCL8*, *CCL2*, *SPP1*, and *TNFA;* and lipid processing genes *APOE*, *ABCA1*, *APOA1*, *LCAT* and *LDLR* in 1°RPE cells (**A, C, E,** and **G**) and RF/6A (**B, D, F** and **H**) cells. (mean and S.E.M.; *n =* 3; *, *p <* 0.05, ns: not significant, two way ANOVA, Sidak's multiple comparisons test); siC, control siRNA; siPPARβ/δ, PPARβ/δ siRNA. Quantification of intracellular lipid accumulation after lipid loading followed by incubation with PPARβ/δ agonist, GW0742 or antagonist, GSK0660 in (**I**) 1°RPE. (*, p<0.05, compared to DMSO Control; ** p<0.05, compared to DMSO; ns: not significant, n=3, two way ANOVA, Sidak's multiple comparisons test); Control: DMSO vehicle, αLA: α-linolenic acid, DHA: docosahexaenoic acid, AA: arachidonic acid, and PA: palmitic acid.

Deletion of *PPARβ/δ* expression caused upregulation of collagen type 1A1 (*COL1A1*) and vitronectin (*VTN*) in human primary RPE cells, and no significant effect on expression levels of collagen type 4A4 (*COL4A4*) and fibronectin (*FN1*) (Figure 2A). In RF/6A cells, deletion of *PPARβ/δ* resulted in downregulation of the extracellular matrix genes *COL1A1*, *COL4A4* and *FN1* (Figure 2B). Increased deposition of collagen type 1A1, collagen 4A4 and vitronectin is characteristic of Bruch's membrane and human sub-RPE deposits typically observed in dry AMD [25], while endothelial cells require extracellular matrix molecules such as *COL4A4*, for pericyte recruitment and vessel stabilization during angiogenesis [26, 27]. These results demonstrate selective roles for *PPARβ/δ* in AMD vulnerable cells suggesting it regulates extracellular matrix turnover in RPE cells similar to that reported for dry AMD, yet inhibits an angiogenic phenotype in endothelial cells. Evaluation of the expression of growth factors that regulate vessel stabilization following *PPARβ/δ* knockdown confirmed this variability in AMD vulnerable cells. A significant decrease in the expression of platelet-derived growth factor receptor beta (*PDGFRB*), vascular endothelial growth factor A (*VEGFA*) and transforming growth factor beta 1 (*TGFB1*) in both primary RPE and RF/6A cells transfected with *siPPARβ/δ*, as compared to control *siRNA* (Figure 2, and D) suggests that disruption of *PPARβ/δ* expression in both of these AMD-vulnerable cells leads to an anti-angiogenic environment in the RPE and choroid. Interestingly, receptor knockdown resulted in a downregulation of the expression of the neurotrophic agent, pigment epithelial-derived factor (*PEDF or SERPINF1*) in RPE cells but not in RF/6A cells (Figure 2, and D). Since modulation of the PPARβ/δ pathway has been shown to regulate inflammation *in vitro* and *in vivo*, the effect of *PPARβ/δ* knockdown on the expression of molecular markers of inflammation was also examined [23, 28, 29]. Genetic knockdown of *PPARβ/δ* resulted in the formation of a pro-inflammatory environment in the outer retinal cells, which was evident by the upregulation of inflammatory genes such as, prostaglandin-endoperoxide synthase 2 (*PTGS2*), interleukin-1 beta (*IL1B*), chemokine ligand 2 (*CCL2*) and tumor necrosis factor alpha (*TNFA*) in RPE cells (Figure 2E); and secreted phosphoprotein 1 (*SPP1*) and *TNFA* in RF/6A cells (Figure 2F). Given the role of PPARβ/δ in regulating lipid processing pathways [30], the expression of genes involved in lipid metabolism and previously shown to be altered in AMD was examined. Increased expression of apolipoprotein E (*APOE*), A (*APOA*) and low density lipoprotein receptor (*LDLR*) in human RPE cells (Figure 2G), along with decreased expression of several lipid transfer genes in choroidal endothelial cells (Figure 2H) following *PPARβ/δ* knockdown was observed. Extracellular and intracellular accumulation of lipids and lipofuscin are characteristics of dry AMD. Good animal models demonstrating significant lipid accumulation in Bruch's membrane and/or deposits, and not requiring aging mice for long periods of time are currently not available. Therefore, in lieu of that, we examined the effect of activating or antagonizing PPARβ/δ in an *in vitro* culture model of lipid-loaded RPE cells. Ligand activation of PPARβ/δ resulted in a significant decrease in RPE lipid accumulation (Figure 2I), suggesting a potential therapeutic avenue to pursue in the treatment of early dry AMD, in which removal of extra- and intra-cellular lipids is a goal. Collectively, these data suggest that though PPARβ/δ drives several of the pathogenic pathways associated with AMD development, it may have selective detrimental and beneficial effects in AMD vulnerable cells. To determine the role of *PPARβ/δ* on the posterior eye, the ocular phenotype of wild-type (*Pparβ/δ*^+/+^) and *Pparβ/δ*-null (*Pparβ/δ*^−/−^) mice was examined.

### Aged *Pparβ/δ*^−/−^ mice exhibit several phenotypic features of dry AMD

**Figure 3:**
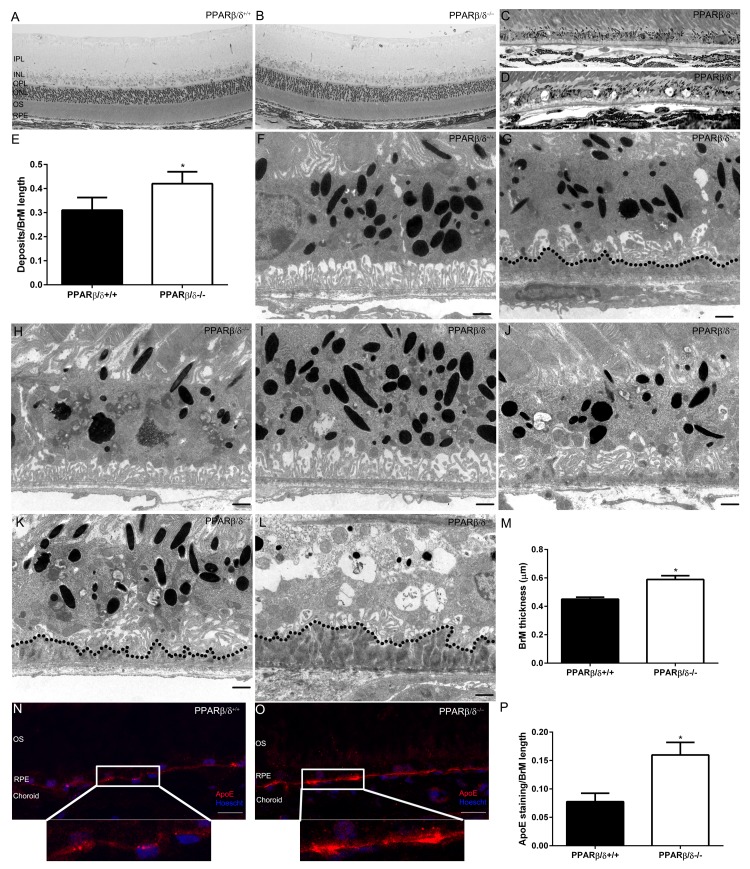
18-month old *Pparβ/δ^−/−^* mice exhibit dry-AMD pathology Toluidine blue stained images of plastic sections from 18-month old (**A**) *Pparβ/δ*^+/+^ and (**B**) *Pparβ/δ^−/−^* mice displaying retinal layers (IPL: Inner plexiform layer, INL: Inner nuclear layer, OPL: Outer plexiform layer, ONL: Outer nuclear layer, OS: photoreceptor outer segments, RPE: Retinal pigment epithelium). Toluidine blue stained images of the outer retina of (**C**) *Pparβ/δ*^+/+^ and (**D**) *Pparβ/δ^−/−^* mice, which have sub-RPE deposits (Scale bar = 10 μm). (**E**) Quantification of deposits per Bruch's membrane (BrM) length in plastic sections (mean and S.E.M., n=10 images/animal, n=4/genotype, * p<0.05). Electron micrographs of RPE/Bruch's membrane/choroidal junction in 18-mo-old *Pparβ/δ*^+/+^ mice display (**F**) normal RPE morphology (**G**) with some age related deposits (dotted line), whereas 18-mo-old *Pparβ/δ^−/−^* mice show (**H**) RPE hypo-pigmentation, (**I**) hyper-pigmentation and (**J**) abnormal basal infoldings with thin sub-deposits, (**K**) loss of basal infoldings with thin and (**L**) thick sub-RPE deposits (dotted line). (**M**) Quantification of Bruch's membrane thickness in electron micrographs of *Pparβ/δ*^+/+^ and *Pparβ/δ^−/−^* mice (*n* = 10 images per mouse, *n* = 4 mice per genotype, two tailed t-test). Scale bars in panels F-L: 1 μm. Images of apolipoprotein E (apoE; red) stained sections from (**N**) *Pparβ/δ*^+/+^ and (**O**) *Pparβ/δ^−/−^* mice (Scale bar: 20 μm). Nuclei are stained with Hoescht (blue). (**P**) Quantification of ratio of apoE stained regions/Bruch's membrane length (mean and S.E.M., n=4 per group, * *p* < 0.05, two tailed t-test).

Gene specific differences in the weights of 18-month old *Pparβ/δ*^+/+^ and *Pparβ/δ*^−/−^ mice were not observed (Figure SF3). The overall architecture of the retina and RPE/choroid was evaluated and no differences in the morphology of the inner retina and/or thickness of the inner and outer nuclear layers were found (Figure SF4). In the outer retina, thin patchy sub-RPE deposits were observed in both 18-month old *Pparβ/δ*^+/+^ and *Pparβ/δ*^−/−^ mice with varying degrees of thickness and length (Figure 3, A, B, C, and D). Detailed quantification of the length of deposit relative to length of Bruch's membrane revealed deposits were present at a higher frequency in *Pparβ/δ*^−/−^ mice compared to *Pparβ/δ*^+/+^ mice (ratio of deposits/BrM length: 0.42 ± versus 0.31 ± for *Pparβ/δ*^−/−^ and *Pparβ/δ*^+/+^ mice, respectively; Figure 3E; 100% of *Pparβ/δ*^−/−^ mice developed deposits, while only 25% of *Pparβ/δ^+/+^* mice developed deposits). Transmission electron microscopy was used for further detailed analysis of the ultra-structure of the RPE-choroid complex. Eighteen-month old *Pparβ/δ*^+/+^ mice exhibited normal RPE morphology with organized basal infoldings (Figure 3F), along with some thin continuous “age-related” sub-RPE deposits (Figure 3G). By contrast, eyes from age-matched *Pparβ/δ*^−/−^ mice exhibited regions with both normal and abnormal RPE morphology. Specifically, the RPE degenerative changes observed included hypo- and hyper-pigmentation (Figure 3, H, I, J, K and L), loss of basal infoldings (Figure 3, J, K and L and L), thickened Bruch's membrane (0.59 ± versus 0.45 ± for *Pparβ/δ*^−/−^ and *Pparβ/δ*^+/+^ mice, respectively; Figure 3M), and a higher frequency of continuous thin and thick sub-RPE deposits (Figure 3, J, K, L and S5) compared to age-matched *Pparβ/δ*^+/+^ mice. Gene specific differences in the morphology and overall structure of the choriocapillaries, including fenestration of the vessels, were not noted (Figure SF5). Given the observed increase in *APOE* expression following *PPARβ/δ* knockdown *in vitro*, apoE protein deposition was determined by immunolocalization in retinal sections. ApoE immunoreactivity was found to be increased within Bruch's membrane and sub-RPE deposits in *Pparβ/δ*^−/−^ mice compared to *Pparβ/δ*^+/+^ controls (Figure 3, N, O and P). RPE autofluorescence, which reflects lipofuscin accumulation, was also examined and found to be significantly higher throughout retinal sections of *Pparβ/δ*^−/−^ mice as compared to age-matched *Pparβ/δ*^+/+^ mice (Figure SF6). Interestingly, evaluation of visual function revealed that the average electroretinographic (ERG) responses from 14-16 month old dark-adapted *Pparβ/δ*^−/−^ and *Pparβ/δ*^+/+^ controls displayed identical dark-adapted a-wave responses, but a slight increase in sensitivity of b-wave amplitudes compared to *Pparβ/δ*^+/+^ mice (Figure SF7 and B). Examination of the localization of markers for inner retinal cells including photoreceptors and bipolar cells by immunohistochemistry failed to demonstrate any gene specific differences (Figure SF7 and D). Overall, these data highlight that PPARβ/δ is essential for the outer retina at the level of the RPE cells, and may regulate sub-deposit formation and RPE degenerative changes as seen in the early stages of dry AMD.

### PPARβ/δ promotes laser-induced CNV

**Figure 4:**
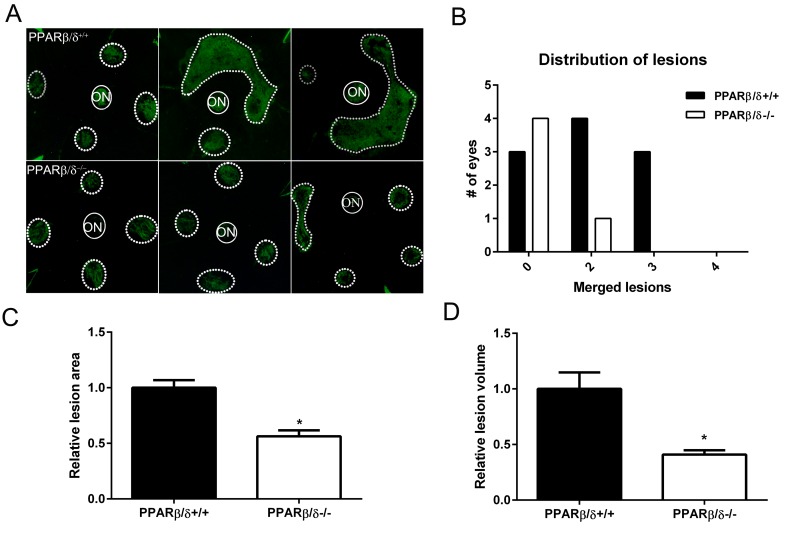
Genetic disruption of *PPARβ/δ* attenuates laser-induced CNV (**A**) Choroidal flat-mounts were prepared from 18–20 month-old laser-induced CNV mice (*Pparβ/δ*^+/+^, *n =* 10 eyes, 40 lesions; *Pparβ/δ^−/−^, n=5* eyes, 20 lesions) and stained with isolectin GS-IB_4_ (ON, optic nerve). Representative images from three eyes/genotype are shown to demonstrate individual and merged CNV lesions. Dotted and solid line circles demarcate lesions and optic nerves, respectively. (**B**) Distribution of number of eyes with individual versus merged lesions in *Pparβ/δ*^+/+^ and *Pparβ/δ^−/−^*. (**C**) Relative lesion area/animal was measured using ImageJ (mean and S.E.M.; *p < 0.01, two tailed t-test). (**D**) Relative lesion volume/animal (mean and S.E.M. for each group; *p < 0.05, two tailed t-test).

Since *PPARβ/δ* knockdown in both RPE and choroidal endothelial cells *in vitro* resulted in an anti-angiogenic phenotype, the role of PPARβ/δ in development of CNV *in vivo* was examined. No evidence of spontaneous CNV or overt vascular changes were observed in aged *Pparβ/δ*^−/−^ mice. However, laser-induced CNV lesions in 18-20 month old *Pparβ/δ*^−/−^ were smaller as compared to those observed in *Pparβ/δ*^+/+^ mouse eyes (Figure 4, A, B and C).

Additionally, examination of the distribution of the lesions in flatmounts of the posterior eye cups revealed a genotype-dependent difference in the number of mouse eyes with single versus merged lesions. Specifically, while *Pparβ/δ*^+/+^ mice presented with two or three merged lesions, no eyes from the *Pparβ/δ*^−/−^ cohort presented with three merged lesions, and only one eye displayed two merged lesions (Figure 4B). Quantitatively, measurement of CNV lesion volume determined from z-stacks and three-dimensional reconstructions confirmed a significantly smaller volume in *Pparβ/δ*^−/−^ mice compared to *Pparβ/δ*^+/+^ mice (Figure 4D). These data demonstrate a functional role for the *PPARβ/δ* gene in angiogenesis and the development of CNV lesions.

### *PPARβ/δ* regulates extracellular matrix deposition and immune cell infiltration in CNV lesions

**Figure 5:**
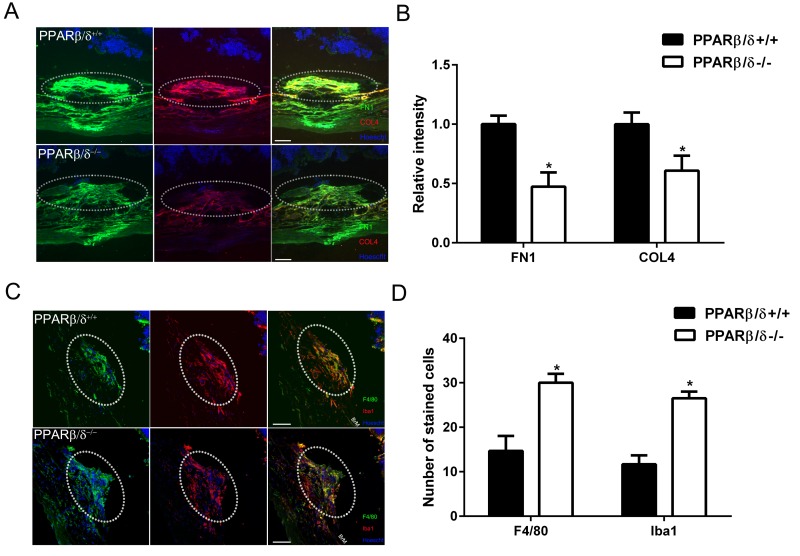
PPARβ/δ regulates extracellular matrix deposition and immune-cell infiltration in CNV lesions (**A**) FN1 (green) and COL4 (red) immunolocalization in CNV lesions of *Pparβ/δ*^+/+^ and *Pparβ/δ^−/−^* mice (dotted oval demarcates the lesion area; nuclei are stained blue with Hoechst; representative images are shown; scale bar = 50 μm). (**B**) FN1 and COL4 staining intensity was quantified in the CNV lesions of *Pparβ/δ*^+/+^ and *Pparβ/δ^−/−^* mice using ImageJ (mean and S.E.M.; *n =* 3/group; ^*^*p < 0.01, two tailed t-test). (**C**) Laser CNV lesions from *Pparβ/δ^−/−^* mice display a higher number of F4/80 (green) and Iba1 (red) immunopositive cells (dotted oval demarcates the lesion area; nuclei are stained blue with Hoechst; representative images are shown; scale bar = 50 μm). (**D)** The numbers of F4/80^+^ and Iba1^+^ cells in the CNV lesions of *Pparβ/δ*^+/+^ and *Pparβ/δ^−/−^* mice were counted using ImageJ (mean and S.E.M.; *n =* 3/group; *p < 0.01, two tailed t-test).

PPARb/d has been shown to regulate extracellular matrix and inflammation [31]. With this in mind, extracellular matrix deposition and immune cell localization within CNV lesions from aged *Pparβ/δ*^+/+^ and *Pparβ/δ*^−/−^ mice were characterized by examining cross sections of the retina/RPE/choroid. Collagen type 4 (COL4) and fibronectin (FN1) are known components of Bruch's membrane, sub-RPE deposits [32-34], and associated with CNV lesions [33]. Furthermore, fibronectin is known to be produced in response to vessel injury [35]. Quantitatively, significantly lower staining intensity for both COL4 and FN1 in CNV lesions was measured in aged *Pparβ/δ*^−/−^ mice compared to age-matched *Pparβ/δ*^+/+^ controls (Figure 5, A and B). As mentioned earlier, PPARβ/δ is known to influence inflammation [31], and this is one of the major pathways that regulates the pathogenesis of AMD [29, 36]. Thus, cross sections of the retina/RPE/choroid containing CNV lesions were probed with antibodies to ionized calcium binding adaptor molecule 1 (Iba1), which labels microglial cells and macrophages, and adhesion G protein-coupled receptor E1, also known as F4/80, which labels mature macrophages (Figure 5 C and S8). Interestingly, a significant increase in the localization of both F4/80^+^ and Iba1^+^ cells within the neovascular lesions of *Pparβ/δ*^−/−^ mice as compared to age-matched *Pparβ/δ*^+/+^ controls was quantified (Figure 5 D and S8). This is a noteworthy observation given the distinct phenotypes that macrophages can differentiate into: 1) the broadly characterized M1 macrophage, which is reported to contribute to pro-fibrotic and pro-inflammatory phenotypes, and 2) M2 macrophages, which also have 4 different subtypes; [37] characterized as having immunosuppressive and tissue remodeling properties [19, 38]. This finding warrants future detailed analysis of the polarity of macrophages in the *Pparβ/δ*^−/−^ CNV lesions. Combined, these results show that the loss of PPARβ/δ expression causes decreased extracellular matrix deposition associated with increased acute immune cell infiltration within small CNV lesions, and may reflect decreased fibrosis modulated by PPARβ/δ.

### Pharmacological antagonism of PPARβ/δ inhibits endothelial cell migration and tube formation *in vitro*

**Figure 6:**
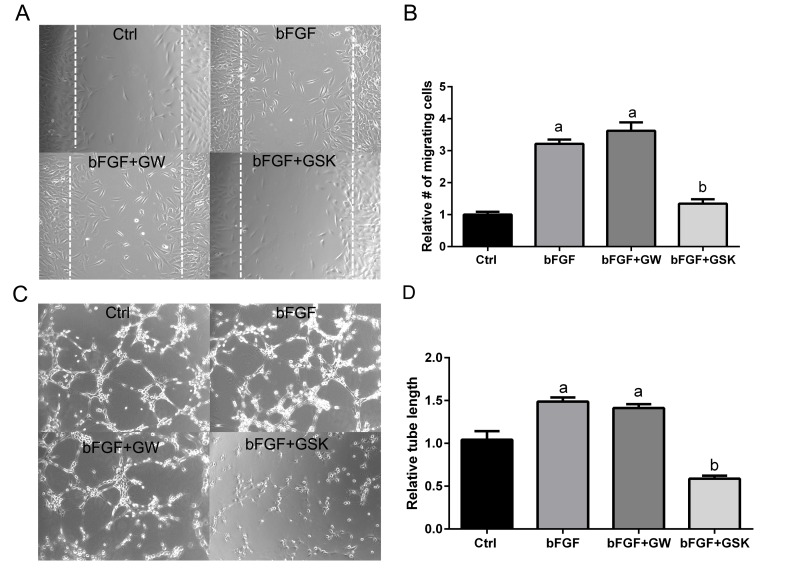
Antagonism of PPARβ/δ blocks endothelial cell migration and tube formation (**A**) The effect of ligand activation or antagonism of PPARβ/δ on migration of RF/6A cells was analyzed in a bFGF induced wound-healing assay (*n =* 3, representative images at *t* = 36 hours are shown); dotted lines demarcate the boarders of the scrape wound. Ctrl: media only, bFGF: basic fibroblast growth factor (10 μg/ml), bFGF+GW: bFGF plus GW0742 (10μM), bFGF+GSK0660: bFGF plus GSK0660 (10 μM). (**B**) The cells migrating into the wound were counted using ImageJ (mean and S.E.M.; *n =* 3; a, *p <* 0.01 relative to Ctrl; b, *p* < 0.01 relative to bFGF, one way ANOVA, Tukey's multiple comparisons test). (**C**) The effect of ligand activation or antagonism of PPARβ/δ on bFGF-induced tube formation in RF/6A cells was analyzed by an angiogenesis assay in Geltrex™ (*n =* 3; representative images at *t* = 3 hours are shown). Ctrl: media only, bFGF: basic fibroblast growth factor (10 μg/ml), bFGF+GW: bFGF plus GW0742 (10 μM), bFGF+GSK0660: bFGF plus GSK0660 (10 μM). Suramin, an inhibitor of tube formation, was used as a negative control (data not shown). (**D**) Quantification of tube length in Geltrex™ using ImageJ (mean and S.E.M.; *n =* 3; a, *p <* 0.01 relative to Ctrl; b, *p* < 0.01 relative to bFGF, one way ANOVA, Tukey's multiple comparisons test).

Cellular organization is a central process in neovascularization, which involves endothelial cell migration and tube formation [39, 40]. Since disruption of the *PPARβ/δ* gene caused smaller CNV lesions *in vivo*, whether pharmacological antagonism of PPARβ/δ could inhibit angiogenesis, using functional assays measuring endothelial migration and tube formation, was determined. Basic fibroblast growth factor (bFGF) was used to induce migration in a scrape wound assay in cultures of choroidal endothelial cells in the presence of the PPARβ/δ agonist, GW0742 or the PPARβ/δ antagonist GSK0660 (Figure 6A). Choroidal endothelial cell migration into the wound was inhibited by antagonism of PPARβ/δ with GSK0660 (Figure 6, A and B), while ligand activation of PPARβ/δ with GW0742 did not influence cell migration. The effect of ligand activation and pharmacological antagonism of PPARβ/δ on the ability of endothelial cells to form a three-dimensional network, indicative of vascular morphogenesis was also assessed (Figure 6C). Similarly, pharmacological antagonism of PPARβ/δ with GSK0660 inhibited tube formation (tube length) in choroidal endothelial cells compared to control and growth factor induced tube formation, whereas ligand activation of PPARβ/δ did not affect tube formation (Figure 6, C and D). In cell viability assays no significant differences were seen between control and PPARβ/δ agonist or antagonist-treated endothelial cells, indicating that the changes in cell migration and tube formation are not due to PPARβ/δ agonist or antagonist-induced cell-death (Figure SF9).

### Pharmacological antagonism of PPARβ/δ attenuates CNV lesions *in vivo*

**Figure 7:**
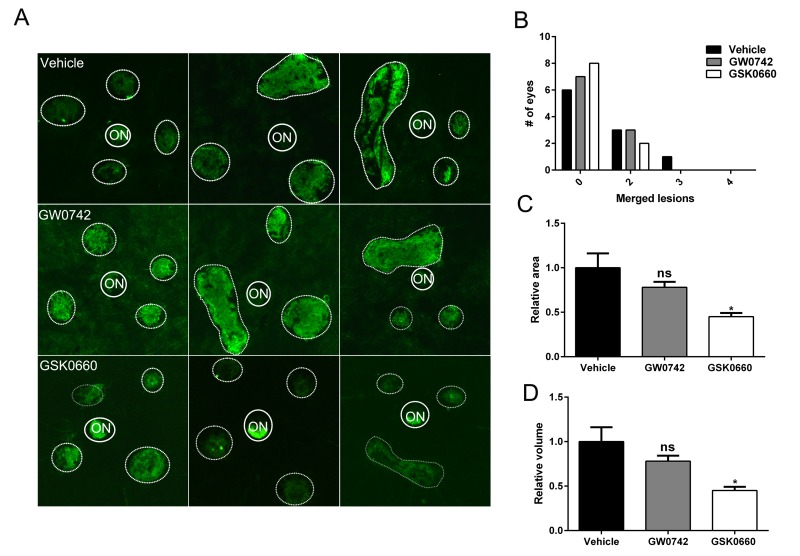
Antagonism of *PPARβ/δ* pathway attenuates CNV (**A**) Choroidal flat-mounts were prepared from 12-13 month-old C57BL/6J (*Pparβ/δ*^+/+^) mice subjected to laser CNV and treated with vehicle control (1% DMSO in saline), GW0742 (0.5 mg/kg/day, i.p.), and GSK0660 (1 m/kg/day, i.p.) and stained with isolectin-GS-IB_4_ (n=10 eyes/group, 40 lesions; ON, optic nerve). Representative images from three eyes/treatment are shown to demonstrate individual and merged CNV lesions (dotted and solid line circles demarcate lesions and ONs, respectively). (**B**) Distribution of number of eyes with individual versus merged lesions in vehicle control, GW0742 and GSK0660. (**C**) Relative lesion area/animal was measured using ImageJ (n=10 eyes per group, mean and S.E.M.; *p < 0.01, one way ANOVA, Tukey's multiple comparisons test). (**D**) Relative lesion volume/animal (n=10 eyes per group, mean and S.E.M. for each group; *p < 0.05. ns: not significant, one way ANOVA, Tukey's multiple comparisons test).

**Figure 8:**
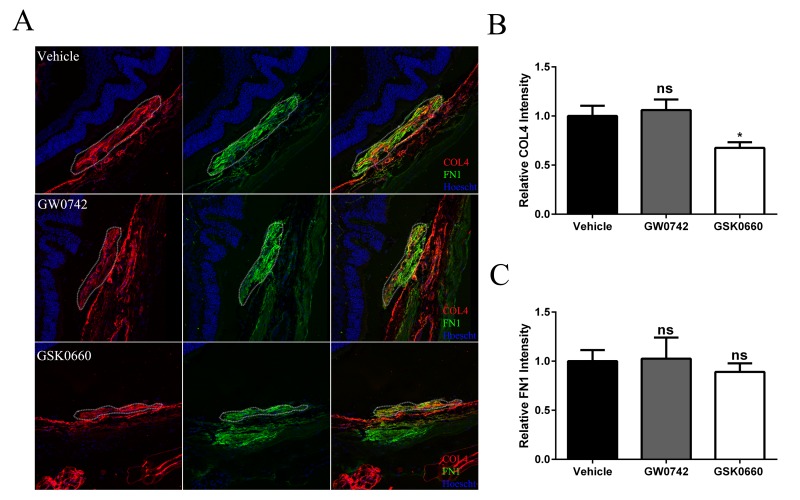
Antagonism of *PPARβ/δ* decreases accumulation of collagen type 4 in CNV lesions (**A**) FN1 (green) and COL4 (red) immunolocalization in CNV lesions of mice treated with vehicle control (1% DMSO in saline), GW0742 (0.5mg/kg/day, i.p.), or GSK0660 (1m/kg/day, i.p.) (dotted oval demarcates the lesion area; nuclei are stained blue with Hoechst; representative images are shown; scale bar = 50 μm). (**B**) COL4 and (**C**) FN1 staining intensity was quantified in the CNV lesions using ImageJ (Mean and S.E.M.; *n =* 3/group; *p < 0.01; ns: not significant, one way ANOVA, Tukey's multiple comparisons test).

The therapeutic potential of inhibiting PPARβ/δ activity on CNV formation in 12-13 month old aged *Pparβ/δ*^+/+^ mice following experimentally induced laser injury to the back of the eye was examined. Mice were treated with vehicle control, GW0742 (0.5 mg/kg/day) or GSK0660 (1 mg/kg/day) (Figure 7A). The distribution, area, and volume of the lesions in flatmounts of posterior eye-cups stained with isolectin GS-IB_4_ were assessed. A treatment dependent difference in the distribution of merged lesions was observed. Antagonism of PPARβ/δ with GSK0660 caused a higher proportion of individual neovascular lesions as compared to vehicle or agonist treated mice (Figure 7B). Additionally, the area and volume of the lesions from animals treated with the PPARβ/δ antagonist were significantly lower relative to vehicle control and PPARβ/δ agonist treated mice (Figure 7, C and D). Finally, examination of cross-sections of the retina/RPE/choroid containing CNV lesions revealed decreased deposition of COL4 within the lesions of mice treated with the PPARβ/δ antagonist, while no difference was observed in the distribution of FN1 (Figure 8, A, B and C). Interestingly, this acute treatment with agonist and antagonist did not effect the distribution of Iba1^+^ and F4/80^+^ cells within the neovascular lesion of aged *Pparβ/δ*^+/+^ mice (data not shown). Noteworthy, this short-term treatment with PPARβ/δ agonist or antagonist, also, did not affect the integrity of the RPE tight junctions (Figure SF010).

Overall, these results are consistent with the *in vitro* studies and demonstrate that inhibition of PPARβ/δ activity results in a less severe CNV phenotype and may therefore be beneficial in attenuating lesion formation *in vivo*.

## DISCUSSION

**Figure 9:**
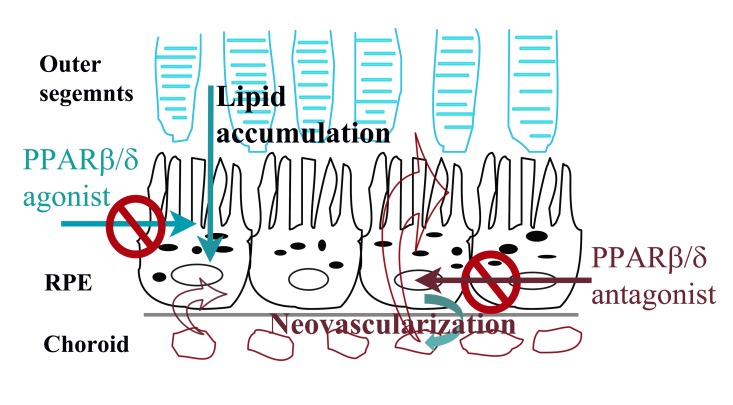
Summary model of selective regulation of dry and wet AMD related pathogenic pathways by PPARβ/δ in AMD vulnerable cells Ligand activation of PPARβ/δ inhibits lipid accumulation by RPE cells and may have therapeutic effect in dry-AMD. In contrast, antagonism of PPARβ/δ inhibits neovasculariza-tion possibly by regulating inflammation and fibrosis in outer-retina.

These studies demonstrate for the first time a role of PPARβ/δ in regulating different aspects of extracellular matrix turnover, angiogenesis, inflammation, and lipid processing in the eye. PPARβ/δ affects the RPE and choroidal endothelium differentially, selectively impacting the development of several fundamental AMD phenotypes. These data show that PPARβ/δ activity is functionally important in RPE and choroidal endothelial cell models systems; cells that are compromised during the initiation and progression of AMD. Knockdown of PPARβ/δ expression led to upregulation of extracellular matrix gene expression in primary RPE cells but a downregulation in choroidal endothelial cells. Additionally, the observed PPARβ/δ-dependent downregulation of the expression of factors critical for angiogenesis, including VEGFA, PDGFRB and TGFB, in both cell populations, supports the hypothesis that PPARβ/δ regulates signaling pathways important in development of neovascular lesions. Consistent with the *in vitro* studies, a significant decrease in area and volume of laser induced neovascular lesions was observed in both aged *Pparβ/δ*^−/−^ mice and in *Pparβ/δ*^+/+^ mice following pharmacological antagonism of the receptor. In contrast to these findings, morpho-logical characterization of the ocular phenotype of aged *Pparβ/δ*^−/−^ mice revealed the exacerbation and development of several features of the early dry AMD phenotype including continuous sub-RPE deposits, increased RPE autofluorescence, Bruch's membrane thickening, RPE pigmentary changes and disorganized basal infoldings. These *in vivo* results are supported by *in vitro* data demonstrating increased dysregulation of extracellular matrix molecules following PPARβ/δ knockdown in human RPE cells. Collectively, these studies illustrate, for the first time, cell-specific effects of PPARβ/δ in two populations of AMD vulnerable cells (Figure 9). Our findings correlate with the concept of selective modulation of PPARs and other nuclear receptors such as the estrogen receptor, in which ligand binding may lead to differential gene expression and biological responses in different cells and tissues [41, 42]. Additionally, part of the variability observed in the eye, may be due to differential expression of PPARβ/δ in the RPE versus choroidal endothelial cells (Figure 1A); differential expression of the receptor co-regulatory proteins including co-activators and co-repressors; differential binding affinities of the co-regulatory proteins; and/or varying receptor conformational changes induced by endogenous ligand binding in epithelial versus endothelial cells [43]. This is an area of ongoing investigation.

The increased influx of immune cells within the neovascular lesion in the absence of PPARβ/δ expression is an interesting finding that requires further investigation and may be clarified with full characterization of the polarization status of these immune cells. Recently, PPARβ/δ has been shown to be involved in alternative activation of macrophages in mice [44]. Reportedly, both IL-13 and IL-4 induce expression of PPARβ/δ and modulate the ability of adipose tissue and liver macrophages to transition to the M2 phenotype. It has also been shown that PPARβ/δ coordinates the immune phenotype of alternatively activated macrophages, both *in vitro* and *in vivo* [45]. These reports suggest that the PPARβ/δ regulates macrophage polarization in animal models of liver-injury and atherosclerosis. However, it is important to note that to date, no one has critically examined the specific subtype of M2 macrophages actually regulated by PPARβ/δ, and since different subtypes have different functions [37], the precise effect of this particular change requires further investigation. Based on these studies it is plausible that the population of Iba1^+^ cells observed in the CNV lesions of *Pparβ/δ*^−/−^ mice are transient and may express markers of the alternatively activated macrophage phenotype, which are involved in immunosuppression and tissue remodeling [19, 38]. CNV lesions in *Pparβ/δ*^−/−^ mice also displayed a reduced deposition of COL4 and FN1 in the lesion supporting a decrease in fibrosis. This is a major factor in responsiveness to anti-VEGF treatment for neo-vascular AMD [46, 47]. To our knowledge this is the first study to report the involvement of the PPARβ/δ pathway in CNV formation.

To date, no differences in *PPARβ/δ* expression between normal and AMD cohorts have been reported. This may be due to the fact that evaluating the activity of PPARβ/δ along with expression of co-factors and specific target genes is more informative than measuring expression of the receptor in silos. Nevertheless, the purported role of PPARβ/δ in other tissues [13, 48-50] in combination with our data from *PPARβ/δ* knockdown *in vitro*, and genetic ablation *in vivo,* gave rise to the hypotheses that pharmacologic targeting of PPARβ/δ may therapeutically improve choroidal neovascularization and lipid accumulation. To test our first hypothesis, the postulate that pharmaco-logic antagonism of PPARβ/δ could therapeutically improve choroidal neovascularization, the effect of ligand activation and pharmacological antagonism of PPARβ/δ on endothelial migration and tube formation in the choroidal endothelial cell line, RF/6A was determined. Pharmacological antagonism of PPARβ/δ activity inhibited choroidal endothelial cell migration as well as tube formation in a three-dimensional matrix. By contrast, ligand activation of PPARβ/δ had no effect in these angiogenesis assays. These results are consistent with previous studies by others using human retinal microvascular endothelial cells and a rat model of oxygen-induced retinopathy [51]. Modeling retinal neovascularization, it was shown that pharmacological antagonism of PPARβ/δ significantly decreased serum-induced retinal endothelial cell proliferation and tube formation in a dose-dependent manner, and *in vivo* decreased retinal neovascularization in young rats. Another study from the same group showed that pharmacological antagonism of PPARβ/δ stabilized tight junctions in retinal microvascular endothelial cells treated with VEGF, and reduced VEGFR1/2 expression, suggesting a role in retinal vascular permeability [52]. The present study examined the effect of ligand activation and pharmacological antagonism of PPARβ/δ on choroidal neovascularization, in which the highly fenestrated choriocapillaris; vasculature supporting the outer retina; grow through Bruch's membrane under the RPE, *in vivo*, using a laser-induced CNV model. Antagonism of PPARβ/δ provided a therapeutic effect on laser-induced lesion formation, whereas ligand activation of PPARβ/δ had no effect, similar to results obtained from our *in vitro* analyses. Interestingly, antagonizing PPARβ/δ activity did not negatively impact the integrity of the RPE cell tight junctions. This may be due to the fact that antagonism of PPARβ/δ was acute rather than long term. Equally likely is that genetic ablation of the receptor (as seen *in vivo* in our mice, but not reported in humans) is detrimental to the RPE cells rather than receptor antagonism itself.

Our second hypothesis supports the notion that it is critical to investigate the therapeutic potential of targeting PPARβ/δ activity in the pathogenesis of dry AMD. Specifically, whether PPARβ/δ activity can be targeted for removal of lipid-rich deposits within Bruch's membrane and sub-RPE deposits should be examined in more detail. Several studies have found that ligand activation of PPARβ/δ inhibits lipid accumulation as well as expression of pro-inflammatory cytokines in THP-1 macrophages and mouse monocytes [53, 54]. Though, the sub-RPE deposits in the aged *Pparβ/δ*^−/−^ mice contain apoE, they did not stain positively for oil red o or filipin and do not appear to contain neutral lipids or cholesterol (data not shown). As proof of concept, using an *in vitro* model of lipid-loaded RPE cells, it was found that ligand activation of PPARβ/δ decreased lipid accumulation. Taken together, these results support the need to test the effect of PPARβ/δ agonists in conditions where there are systemic and/or dietary factors contributing to development of early AMD. Though there is selective modulation of the PPARβ/δ in RPE and choroidal endothelial cells, based on the *in vitro* and *in vivo* data, ligand activation of PPARβ/δ is beneficial to RPE cells and does not appear to have a negative impact on healthy choroidal endothelial cells. Currently there are no FDA-approved PPARβ/δ drugs, but the recent development of highly specific and selective agonists and antagonists [selective PPARβ/δ modulators (SPPARMs)] such as GW501516, GW0742, GSK0660 and GSK3787 have led to improved approaches to study the PPARβ/δ pathway in disease models including obesity and atherosclerosis [55]. In future studies, it will be imperative to construct PPARβ/δ-selective drug-dose response curves to describe cell-selective responses in a quantitative manner and test the suitability of pharmacological agents in long-term studies using aged animal models of AMD that develop lipid accumulation within Bruch's membrane.

This is the first study to report on the PPARβ/δ pathway in RPE and choroidal endothelial cells and its potential role in the pathogenesis of AMD. The results indicate, for the first time, selective modulation of a nuclear receptor in the eye. Specifically, that inhibition of PPARβ/δ activity may successfully attenuate neovascu-lar lesion formation concomitant with decreased expression of extracellular matrix molecules and decreased angiogenic factors, *in vivo,* while activation of PPARβ/δ activity may target lipid accumulation. The results of this treatment may be attributable to PPARβ/δ regulation of several distinct AMD pathogenic pathways: fibrosis, inflammation and lipid metabolism. The *Pparβ/δ*^−/−^ mice also displayed early dry AMD like pathology, establishing these mice as a model to further study the initiation and progression of the early sub-type of the disease. Additional studies should be conducted to study the contribution of other PPARβ/δ regulatory pathways that may influence disease pathology and the therapeutic potential of selectively targeting PPARβ/δ as a means to inhibit inflammation, fibrosis and lipid accumulation (Figure 9).

## MATERIALS AND METHODS

### Cell lines

RF/6A cells, a spontaneously transformed choroidal endothelial cell line derived from the eyes of a rhesus macaque fetus, passages 35–40, and ARPE19 cells, a spontaneously arising human RPE cell line derived from the eyes of a 19 year-old male donor, passages 21–28, were obtained from ATCC (Manassas, VA, USA). Primary cell cultures used included RPE cells (1° RPE) isolated from donor eyes older than 60 years (n=3), collected from the North Carolina Organ Donor and Eye Bank Inc. in less than 6  hours post-mortem and cultured within 24 hours in accordance with the Declaration of Helsinki for research involving human tissue, as previously described [56]. Donor eyes did not reveal any evidence of retinal/RPE changes upon post-mortem evaluation of the posterior eye under a dissecting microscope. Only passages between 4 and 9 of each of the primary RPE cell cultures were used in this study. Final conditions of both ARPE19 and the primary RPE cell cultures were such that cells were post-confluent and demonstrated zonula occludens positive immunoreactivity.

### Transcriptional activation assay

The transcriptional activity of PPARβ/δ was measured using a luciferase-based reporter assay and *PPARβ/δ* target gene expression was quantified using qPCR. Briefly, 50000 human primary RPE cells, ARPE19 cells or RF/6A cells/well were seeded in 24-well plates in phenol red-free medium supplemented with 7.5% charcoal-stripped FBS and cultured overnight. Lipofectin (Invitrogen)-mediated transfection was performed the following day, using plasmids encoding a DR-1 luciferase reporter, CMV-*β* galactosidase or pBSII, as described previously [57, 58]. After overnight culture, cells were transfected with small interfering RNAs (siRNA) (scrambled control siRNA or an siRNA against PPARβ/δ; [100 pmoles per 250,000 cells]); 5 hours after knock-down, the transfected cells were treated with PPARβ/δ agonist and antagonist at doses listed in Table S1. The cells were lysed 24 hours later for luminescence reading. Luciferase (reporter) and *β*-galactosidase [chlorophenol red *β*-d-galactopyranoside (CPRG) as substrate; transfection normalization] activities were measured using a Perkin-Elmer fusion instrument. Concomitantly, cells were treated with these same compounds for RNA isolation and target gene expression studies. All samples were run in triplicate and experiments were performed a minimum of three times.

### siRNA transfection and cell functional assays

siRNAs were used to knock down *PPARβ/δ in vitro.* A control siRNA (siC) for a non-targeting sequence or 5 different PPARβ/δ siRNAs (siPPARβ/δ, siPPARβ/δ1, siPPARβ/δ2, siPPARβ/δ3, siPPARβ/δ4; GE Dharmacon, Lafayette, CO, USA; Table S2) were trans-fected into cells using Lipofectamine RNAiMAX (Invitrogen, Grand Island, NY, USA) and tested to account for non-specific targeting of the siRNA (Figure SF011). Briefly, complexes of siRNA (100 pmoles per 250,000 cells) and RNAiMAX in OPTI-MEM were added to six-well cell-culture plates according to the manufacturer's protocol. siPPARβ/δ was used in subsequent experiments. ARPE19, 1° RPE or RF/6A cells (250 000 cells/well) were added to each well, along with DMEM/F12 or MEM supplemented with 10% charcoal-stripped fetal bovine serum (CS-FBS) for ARPE19 or 1° RPE, and endothelial cells, respectively. Cells were used 24 hours post-transfection, in cell viability assays, scrape wound migration assays (MEM 1% CS-FBS), tube-formation assays (MEM 1% FBS) and PPAR activity assays (7.5% CS-FBS), as described. RNA was extracted at the indicated time points for quantitative real-time PCR (qPCR). Primer sequences are provided in Table S3.

### RNA Isolation and qPCR

Total RNA isolation from cultured cells, freshly isolated human RPE cells and choroid; RNA quality assessment; cDNA reverse transcription; and qPCR were completed as previously described [59]. The purities of the freshly isolated human RPE cells and choroid from aged donor eyes were determined by measuring the expression levels of RPE specific markers including *BEST1* and *RPE65* and a vascular marker, *PECAM*. Minimal levels of cross contamination were found (Figure SSF12). qPCR was performed using the Bio-Rad CFX96 Realtime PCR Detection System (Bio-Rad). Melt curves for each pair of primers were inspected to confirm a single amplicon. The Ct values were normalized to a housekeeping gene (acidic ribosomal phosphoprotein P0, 36B4). Gene-expression fold changes were calculated using the ΔΔCT method. Primer sequences used were selected from Primer Bank, http://pga.mgh.harvard.edu/primerbank and are present-ed in Supplementary Table S3. The amplification products obtained after qPCR using PPARβ/δ, RXRα, RXRβ, BEST1, RPE65, PECAM and 36B4 primers were run on a 1% agarose gel and visualized with ethidium bromide.

### Adipored assay

AdipoRed™ (Lonza, Walkersville, MD) assay was used to measure intracellular lipid accumulation according to manufacturer's protocol. Briefly, human primary RPE cells were plated in a 96-well plate (10000 cells/well) in DMEM/F12 media with 7.5% charcoal stripped FBS. The cells were treated with different lipids (20μM) namely, α-linolenic acid (α-LA), docohexanoic acid (DHA), arachidonic acid (AA), and palmitic acid (PA) for 48 hours, followed by a 24 hour treatment with GW0742 (10 μM) and GSK0660 (10 μM). The cells were then washed with PBS and treated with AdipoRed™ reagent. After 10 minute incubation, the fluorescence was read at 485 nm excitation and 572 nm emission.

### Animals

Male and female *Pparβ/δ*^+/+^ and *Pparβ/δ*^−/−^ mice on the C57BL/6 background [60], aged (*Pparβ/δ*^+/+^: n=13, 13 females, 3 males; *Pparβ/δ*^−/−^: n=10, 7 females, 3 males) were maintained in a temperature (25°C) and light controlled (12h light/12 h dark) environment and provided standard mouse chow ad libitum. Mice were screened for the confounding retinal degeneration 8 mutation and its absence was confirmed as previously described [56].

### Study approval

The study protocols were approved by the Duke University or Pennsylvania State University Institutional Animal Care and Use Committees. All animal experiments were performed in accordance with the guidelines of the ARVO statement for the Use of Animals in Ophthalmic and Vision Research.

### Immunohistochemistry and morphology

For immunohistochemistry, eyes were fixed in 4% paraformaldehyde and cryopreserved. Specimens were cryosectioned from the superior cup through the optic nerve to the inferior cup in 10 μm increments. Cryosections from the nasal, central and peripheral regions of the eye were probed with antibodies (listed in Supplementary Table S4). Non-specific immuno-staining in sections was blocked with normal serum (Jackson Immunoresearch, West Grove, PA, USA) appropriate to the secondary antibody species. Secondary antibodies were conjugated to AlexaFluor 568 and 488 (Invitrogen). Control slides containing sequential sections were probed with non-immune serum and buffer without primary antibody. Nuclei were stained with Hoechst 33258 (Invitrogen). Images were collected on a Nikon C1 confocal microscope and visualized and processed using Nikon EZ-C1 Free viewer.

### Transmission electron microscopy

For electron microscopy, eyes were fixed in 2% gluteraldehyde, post fixed in 1% osmium tetroxide, and embedded in Spurrs resin. Morphology of the retina/RPE/choroid was studied in 1 μm toluidine blue stained plastic sections. The length of deposits/RPE length was calculated and plotted for *Pparβ/δ*^+/+^ and *PPARβ/δ^−/−^* mice (10 images per mice, n=4 mice per genotype). Incidence of pathology in *PPARβ/δ^−/−^* eyes, including RPE abnormalities (seen in greater than 20% of the eye), presence of sub-RPE deposits, and thickness of Bruch's membrane, were evaluated in electron microscopy thin sections (10 images per mouse, n = 4 mice per genotype).

### Electroretinography (ERG)

ERGs were recorded using the Espion E^2^ system (Diagnosys LLC) as described previously [56]. Briefly, 14-16-mo-old *Pparβ/δ*^+/+^ and *PPARβ/δ^−/−^* mice were dark-adapted for four hours and anesthetized by an i.p. injection of a ketamine/xylazine mixture (85/10 mg/kg). Pupils were dilated with 1% cyclopentolate-HCl and 2.5% phenylephrine, and the mouse body temperature was maintained at 37°C using a water-based warming pad. ERG responses under dark-adapted (“scotopic”) conditions were evoked by a series of nine flashes ranging from 0.0001 cd·s/m^2^ to 100 cd·s/m^2^. For flashes up to 0.1 cd·s/m^2^, responses of 10 trials were averaged. For 0.5 and 1 cd·s/m^2^ flash responses, three trials were averaged. For brighter stimuli, responses to single flashes were recorded without averaging. Light-adapted (“photopic”) ERGs were evoked by a series of six flashes ranging from 0.2 cd·s/m^2^ to 2,000 cd·s/m^2^ whereas rod inputs were suppressed with a steady background illumination of 50 cd/m^2^. Up to 10 trials were averaged for all flash responses. Analysis of a- and b-wave amplitudes was performed as described [56].

### Mouse model of CNV

Laser photocoagulation was performed in cohorts of 18-20 month old *Pparβ/δ*^+/+^ (n=7, all females) and *PPARβ/δ^−/−^* mice (n=4, 3 females, 1 male), as previously described [59]. Briefly, four thermal burns were induced in each eye around the optic nerve, using a slit lamp delivery system. The mice were euthanized 3 weeks after laser treatment and the eyes were harvested for visualization of laser-induced CNV in posterior pole flat-mounts, or cryopreserved for immunohisto-chemistry and morphology experiments. To test the efficacy of PPARβ/δ drugs, 12-13-month old *Pparβ/δ*^+/+^ mice were divided into three cohorts (n=10/cohort, 5 females and 5 males per cohort), vehicle control (1% DMSO in saline), GW0742 (0.5 mg/kg/day, i.p.) [61, 62] and GSK0660 (1 mg/kg/day, i.p.) [63]. Animals were pre-treated with the drugs for 2 days prior to laser CNV induction and euthanized 10 days later. CNV lesion volume, area and size were measured in flat-mounts stained with isolectin GS-IB_4_ to examine vascularity of the neovascular lesion.

### Evaluation of mouse CNV lesions

Following laser CNV, flatmounts of the posterior pole were stained with isolectin GS-IB_4_ Alexa Fluor^®^ conjugate (Life Technologies, Grand Island, NY) according to manufacturer's protocol, to examine vascularity and size of the neovascular lesion, and visualized by a Zeiss Axiolan 2 (Carl Zeiss, Thornwood, NY) fluorescent microscope. Horizontal optical section images of the flatmounts were obtained at 1.50 μm intervals. CNV lesion thickness was measured using Nikon EZ-C1 viewer software. Total area of the CNV lesions per lesion per animal was measured using ImageJ software (developed by Wayne Rasband, National Institute of Health, Bethesda, MD) and the volume of the lesions was measured by running the MeasureStacks script in ImageJ. All measurements were normalized to *Pparβ/δ*^+/+^ or vehicle control in their respective experiment. Cryosections from the lasered eyes were screened for lesions and stained with F4/80 and Iba1 antibodies (Supplementary Table S4). CNV was demarcated and cells staining positive for F4/80 and Iba1 were counted and plotted. To study extracellular matrix deposition, cryosections were stained with FN1 and COL4 antibodies. CNV lesions were demarcated and mean fluorescence intensity of FN1 and COL4 was measured by ImageJ. Mean intensity was plotted.

### Scrape wound migration assay

Following *PPARβ/δ* knock-down in RF/6A cells by siRNA transfection, as described in the main text, 250000 cells were added to each well in six-well plates; 24 hours after knock-down, the cell monolayer was scraped in a direction perpendicular to a horizontal line, using a 1000 μl pipette tip, to create a wound. bFGF (100 ng/ml)-induced cell motility was observed at *t* = 0 and 36 hours post-scraping. The total number of cells migrating into the wound at *t* = 36 were counted using ImageJ and normalized to control siRNA. Similarly RF/6A cells were pretreated with PPARβ/δ agonist, GW0742 or antagonist, GSK0660, at doses listed in Table S1 for 24 hours. It was followed by scrape wound assay. Data were generated from four fields of view/experiment in a total of three biological replicates.

### Tube-formation assay

Tube formation assay was used as a model for angiogenesis. Geltrex™ (Life Technologies, Grand Island, NY, USA) was thawed overnight at 4°C. Using cold pipette tips, 200 μl/well was added in a 12-well plate. The Geltrex solidified into a thin layer after incubation at 37°C for 1 hour. *PPARβ/δ* was knocked down in RF/6A endothelial cells by siRNA transfection, trypsinized after 48 hours and plated onto the Geltrex-coated wells (2.5 × 10^4^ cells/well). Network formation was examined after 3 hours, using an inverted phase-contrast microscope, and quantified as total tube length formed by siPPARβ/δ-endothelial cells normalized to siControl, using ImageJ. Similarly RF/6A cells were pretreated with PPARβ/δ agonist, GW0742 or antagonist, GSK0660, at doses listed in Table S1 for 24 hours followed by scrape wound assay. Four fields of view/experiment were examined, in a total of three biological replicates.

### Cell viability and proliferation assays

Primary human RPE, ARPE19, and RF/6A cells were plated in 96-well plates at different cell densities (5000-, and 10000-cells per well) and treated with DMSO, GW0742 (10μM) and GSK0660 (10μM). Cell viability was measured after four days post-plating using CellTiter-Blue® (Promega, Madison, WI), according to the manufacturer's protocol. The results obtained from 10000-cells/well are reported.

### Lipofuscin Quantification

A Leica Spectral Laser Scanning Confocal Microscope was used to measure autofluorescence throughout the entire RPE layer of 18-mo-old *PPARβ/δ^−/−^* and *Pparβ/δ*^+/+^ cryosections (*n* = 3 sections per mouse, *n* = 3 mice per genotype) as previously described [64]. The three sections were selected randomly from the nasal, central, and temporal regions of the mouse eye. Lambda (λ) scans were performed using a 405-nm laser. Excitation and emission frequencies were measured with a 5-nm-wide band through a spectral range from 422.5 nm to 722.5 nm using serial 30-image scans at ∼10.3-nm intervals. Fluorescent intensities are represented as arbitrary units as defined by the confocal Leica software. The significance of differences in spectra obtained between *PPARβ/δ^−/−^* and *Pparβ/δ*^+/+^ mice was assessed using a two-tailed *t* test, with no variance assumptions.

### Statistics

Statistical methods for data analysis included two-tailed Student's *t*-test and two-way ANOVA, with Sidak's multiple comparison test using GraphPad Prism. Values were considered statistically significant at *p <* 0.05.

## SUPPLEMENTARY MATERIAL FIGURES AND TABLES


