# Supplementary material for: PPARβ/δ selectively regulates phenotypic features of age-related macular degeneration
Source: Aging (Albany NY). 2016 Sep 8;8(9):1952–71. doi: 10.18632/aging.101031 (PMC5076447; doi:10.18632/aging.101031)
Supplement: Supplementary file 1 [file aging-08-1952-s001.pdf]

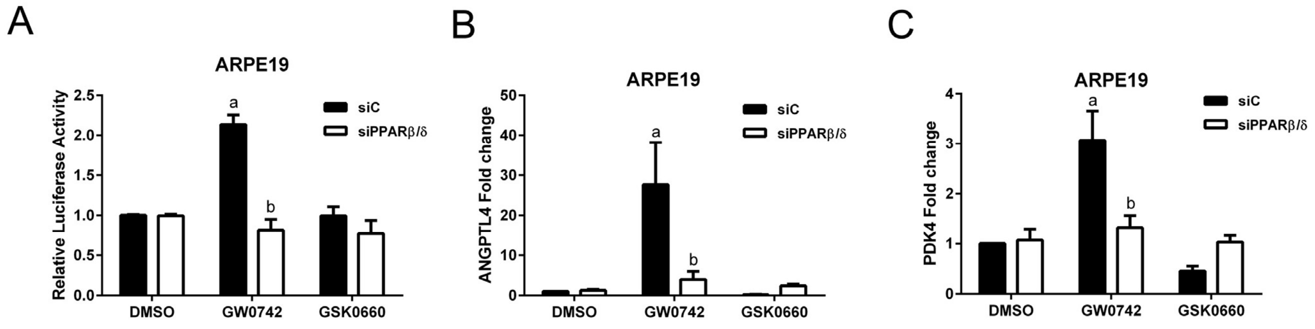

Figure S1. PPARβ/δ signaling pathway is functional in ARPE19 cells. (**A**) PPARβ/δ activity in ARPE19 cells transfected with the DR1 luciferase reporter and siC or siPPARβ/δ; cells were treated with PPARβ/δ agonist, GW0742 or antagonist, GSK0660 or DMSO as control ($n = 3$): a, $p < 0.05$ relative to DMSO treated cells; b, $p < 0.05$ relative to drug+siC treated cells ($p < 0.05$). Expression of (**B**) *ANGPTL4* and (**C**) *PDK4* mRNA in siC and siPPARβ/δ treated ARPE19 cells in response to GW0742, GSK0660, or DMSO as a control ($n = 3$); a: $p < 0.05$ relative to DMSO treated cells; b: $p < 0.05$ relative to drug+siC treated cells.

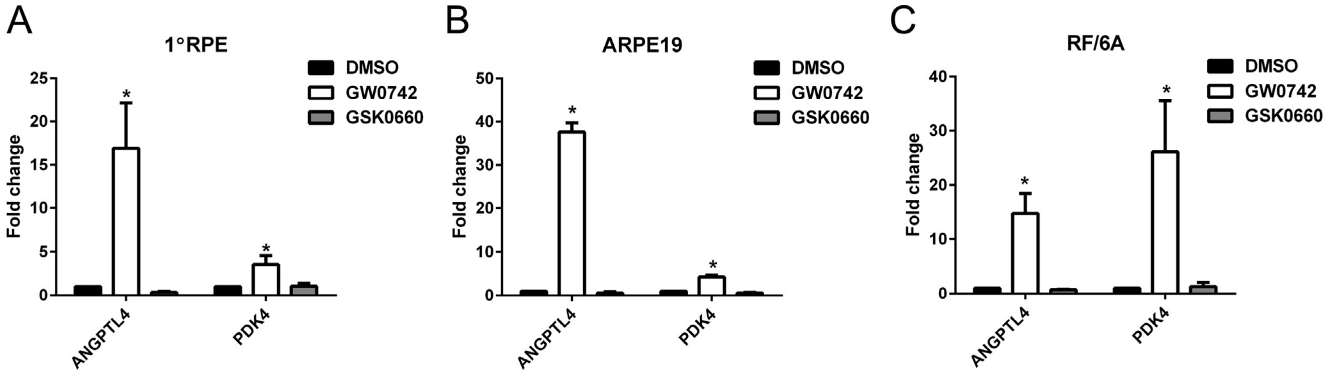

Figure S2. PPARβ/δ agonist and antagonist treatment of AMD vulnerable cells in the absence of transfection differentially effect receptor target gene expression. Human primary RPE (1°RPE); (**A**), ARPE19 (**B**), and RF/6A (**C**) cells were treated with GW0742 (10μM) and GSK0660 (10μM) for 24 hours, using DMSO as control. mRNA levels of the PPARβ/δ target genes *ANGPTL4* and *PDK4* were measured using qRT-PCR (n=3, p<0.05).

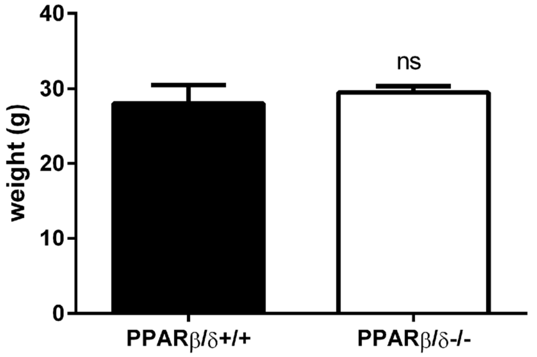

Figure S3. Comparison of body weights of age-matched *Pparβ/δ*$^{+/+}$ and *Pparβ/δ*$^{-/-}$ mice. Average weights of mice from *Pparβ/δ*$^{+/+}$ (n=7) and PPARβ/*δ* $^{-/-}$ (n=4) cohorts. (mean and S.E.M., ns: not significant).

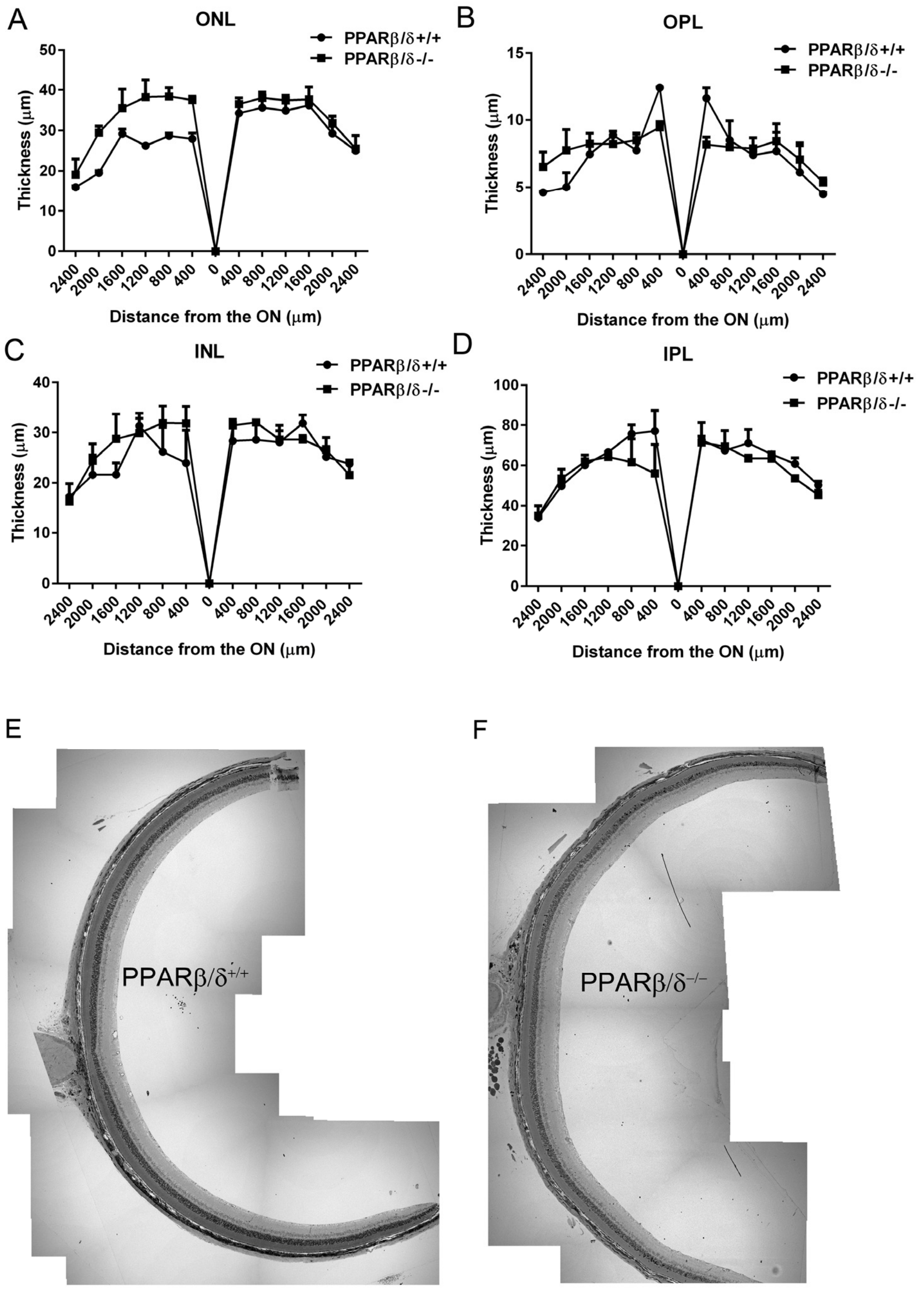

Figure S4. Quantification of thickness of retinal layers of *Pparβ/δ*$^{+/+}$ and *Pparβ/δ*$^{-/-}$ mice. Toluidine blue stained retinal sections were imaged from age matched *Pparβ/δ*$^{+/+}$ (n=3) and *Pparβ/δ*$^{-/-}$ (n=2) mice at 20X from end to end and images were tiled to create complete retinal section images. Six equidistant measurements of (**A**) outer nuclear layer (ONL) (**B**), outer plexiform layer (OPL), (**C**) inner nuclear layer (INL), and (**D**) inner plexiform layer (IPL) towards each end were made from the optic nerve. The thickness of these layers was plotted. Tiled images demonstrating overall retinal morphology of (**E**) *Pparβ/δ*$^{+/+}$ and (**F**) *Pparβ/δ*$^{-/-}$ mice.

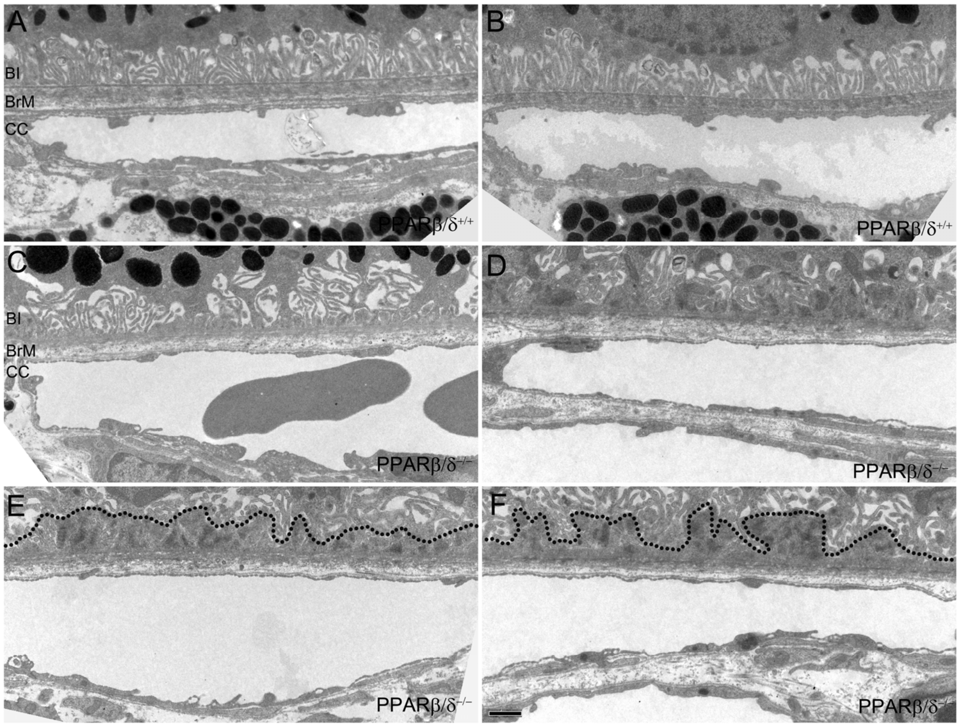

Figure S5. Genetic ablation of *Pparβ/δ* does not affect the integrity of the choriocapillaris. Morphology of the choriocapillaris was examined in electron micrographs. (**A, B**) Normal organization of the basal infolding of the RPE, Bruch's membrane (BrM) and fenestrated choriocapillaries (CC) in *Pparβ/δ*$^{+/+}$ mice (n=4). (**C, D, E, F**) Sub-RPE deposits, disorganized basal infoldings of the RPE above morphologically normal looking fenestrated choriocapillaries. Apical limit of the sub-RPE deposits is outlined by a dotted line. N=8-14 images/mouse, n=4 mice/genotype were examined. Scale bar: 1 µm.

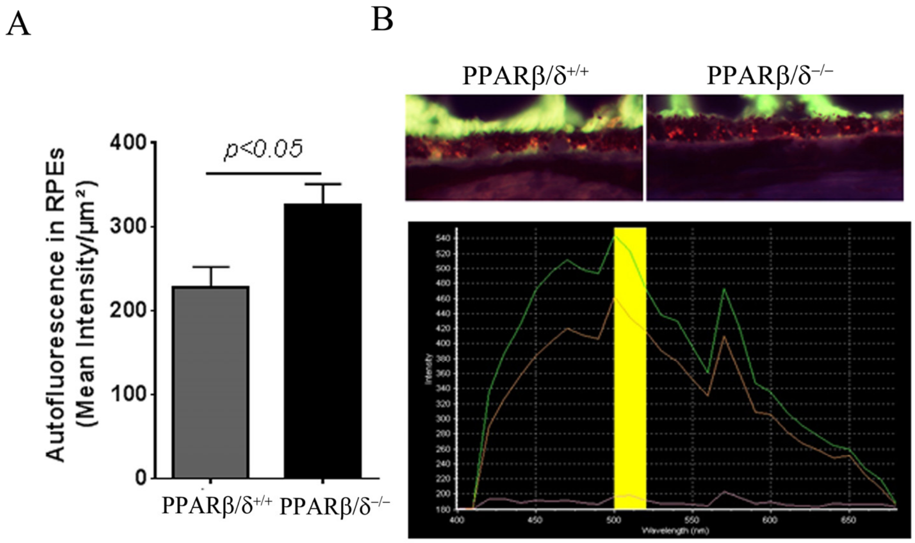

Figure S6. Genetic disruption of *Pparβ/δ* leads to lipofuscin accumulation *in vivo.* RPE autoflourescence was measure in *Pparβ/δ*$^{+/+}$ and *Pparβ/δ*$^{-/-}$ mice. (**A**) Quantification of lipofuscin autoflourescence in RPE from *Pparβ/δ*$^{+/+}$ and *Pparβ/δ*$^{-/-}$ mice. (**B**) Representative images and (**C**) plot of intensity versus wavelength of cryosections from *Pparβ/δ*$^{+/+}$ and *Pparβ/δ*$^{-/-}$ mice.

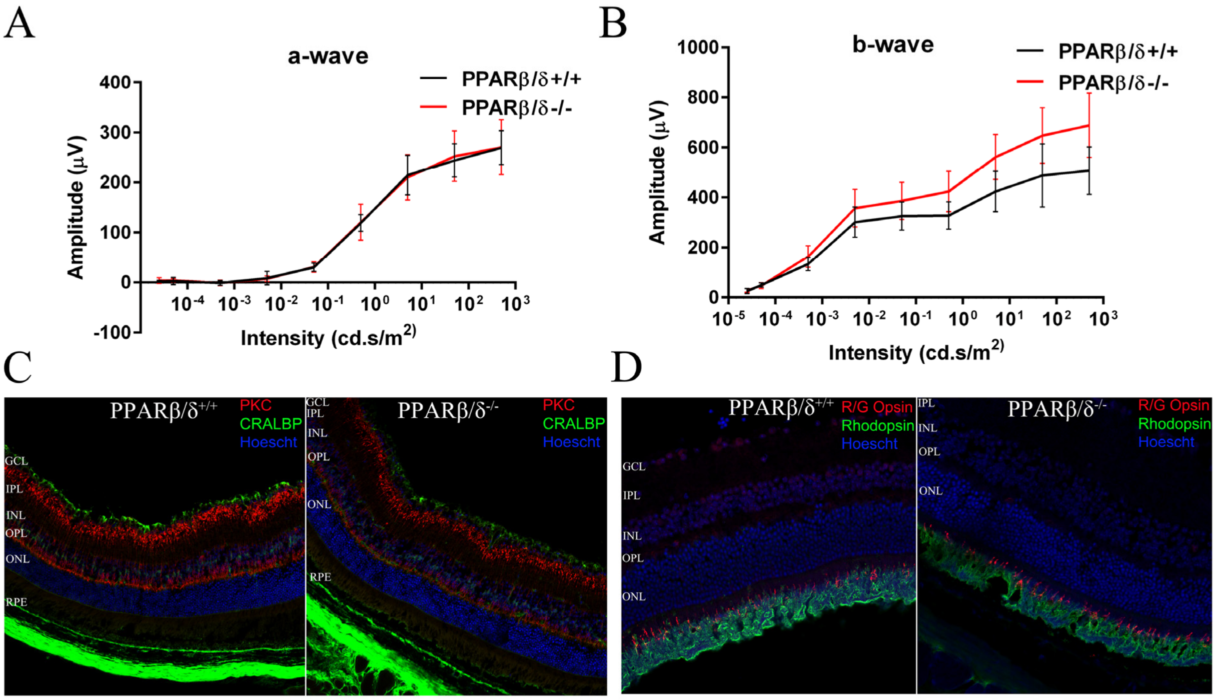

Figure S7. *Pparβ/δ*$^{-/-}$ mice exhibit comparative ERG responses to age-matched *Pparβ/δ*$^{+/+}$ mice. Averaged ERG responses in 14-16 month old dark-adapted *Pparβ/δ*$^{+/+}$ (black) (n=5) and *Pparβ/δ*$^{-/-}$ mice (red) (n=6). Plots of (**A**) dark-adapted a-wave amplitudes and (**B**) b-wave amplitudes as a function of flash intensity. Data points are mean and S.E.M. Immunolocalization of retinal markers in cryosections from 18-month old *Pparβ/δ*$^{+/+}$ and *Pparβ/δ*$^{-/-}$ mice probed with antibodies for (**C**) rod bipolar cells (PKCα, red) and Müller cells (CRALBP, green) and (**D**) red-green cone photoreceptors (R/G opsin, red) and rod photoreceptors (rhodopsin, green). Nuclei are stained with Hoescht (blue).

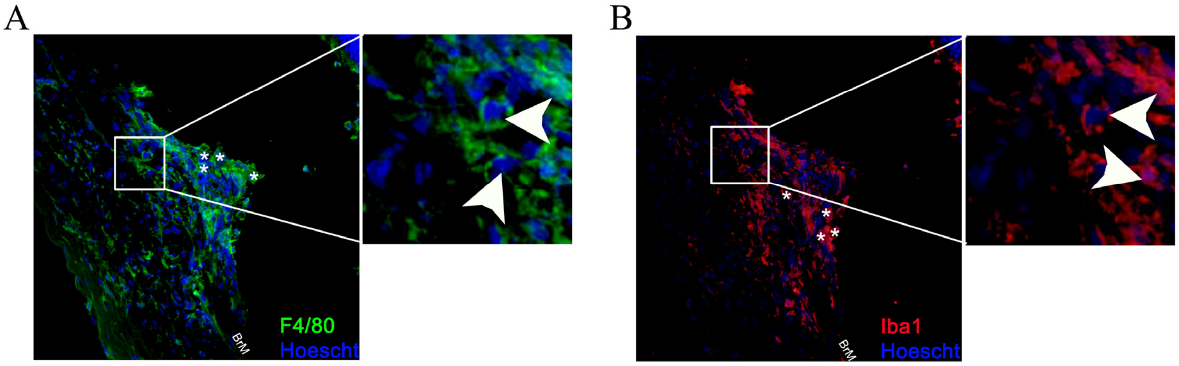

Figure S8. **High magnification view of Iba1 and F4/80 staining in laser CNV lesions.** Laser CNV lesions from *Pparβ/δ*$^{-/-}$ mice display distinct F4/80 (green) (**A**) and Iba1 (red) (**B**) immunopositive cells. White arrowheads depict individual cells stained with F4/80 or Iba1. Asterisks mark some additional examples of positively stained cells.

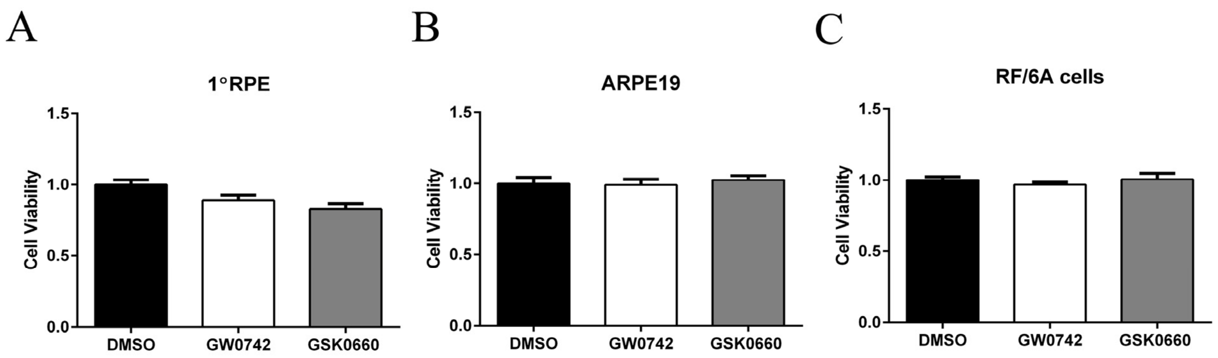

Figure S9. Ligand activation or pharmacological antagonism of PPARβ/δ does not affect cell viability in cell-culture models. Cell viability was measured after a 4 day treatment with the PPARβ/δ agonist, GW0742 or antagonist, GSK0660 in **(A)** primary RPE cells, (**B**) ARPE19 and (**C**) RF/6A cells.

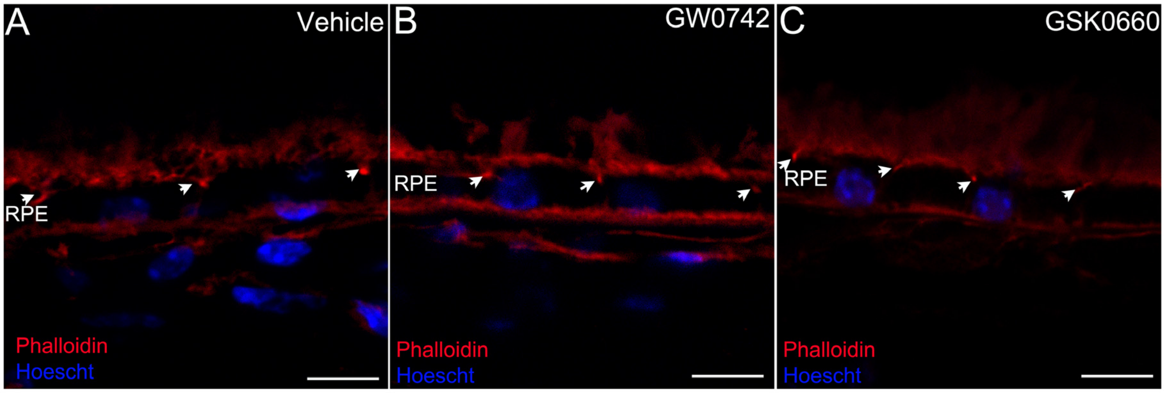

Figure S10. Ligand activation or pharmacological antagonism of PPARβ/δ does not affect RPE tight junctions *in vivo*. Localization of phalloidin (red) in cross-sections from mice treated with (**A**) vehicle control (1% DMSO in saline), (**B**) GW0742 (0.5mg/kg/day, i.p.), or (**C**) GSK0660 (1m/kg/day, i.p.). Apical localization is indicated by white arrows. Hoescht was used to stain nuclei.

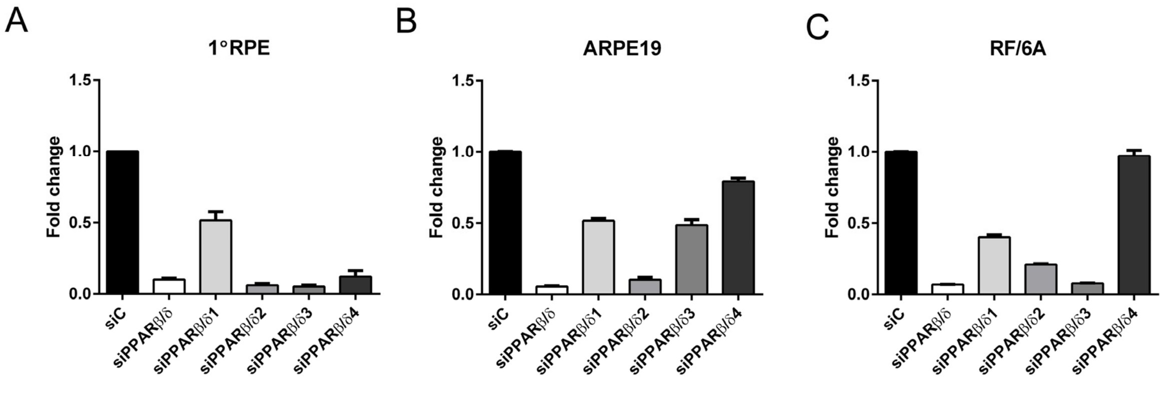

Figure S11. Validation of *PPARβ/δ* knockdown in cell-culture models. Multiple siRNAs targeting PPARβ/δ listed in Table S2 were tested in cell culture models used in this study. RT-PCR was used to measure the expression of *PPARβ/δ* mRNA levels 24 hours after siRNA transfection. Effect of siRNA transfection on *PPARβ/δ* gene expression in (**A**) primary RPE cells, (**B**) ARPE19 and (**C**) RF/6A cells. siC, control siRNA; siPPARβ/δ, PPARβ/δ siRNA.

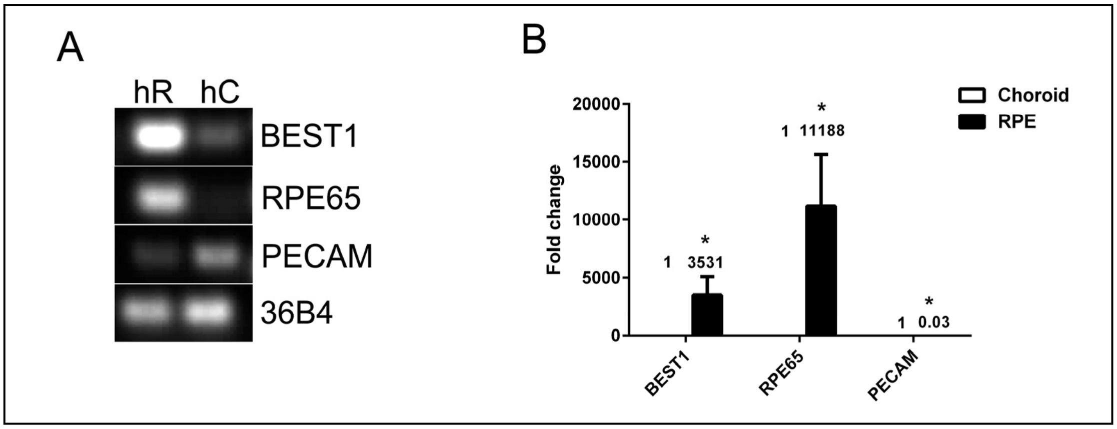

Figure S12. Freshly isolated RPE and choroid from human donor eyes have minimal cross contamination. mRNA expression of *BEST1*, *RPE65* and *PECAM* in freshly isolated RPE cells relative to freshly isolated choroidal cells (**A**, n=3, p<0.05), The mean fold change for each gene is depicted on the graph (**B**).

Table S1. List of drugs, concentrations, and sources.

Drugs	Concentration	Source
**GW0742**	10 μM	Sigma-Aldrich
**GSK0660**	10 μM	Sigma-Aldrich

Table S2. List of siRNAs.

siRNA	Company	Catalog#
**siC**	Qiagen	1027281
**siPPARβ/δ**	Dharmacon	L-003435-00-0005
**siPPARβ/δ1**	Dharmacon	J-003435-06
**siPPARβ/δ2**	Dharmacon	J-003435-07
**siPPARβ/δ3**	Dharmacon	J-003435-08
**siPPARβ/δ4**	Dharmacon	J-003435-09

Table S3. List of primers.

Gene	Primers	Sequence 5'→3' Human
**36B4**	Forward	GGACATGTTGCTGGCCAATAA
	Reverse	GGGCCCGAGACCAGTGTT
**ANGPTL4**	Forward	GTCCACCGACCTCCCGTT A
	Reverse	CCTCATGGTCTAGGTGCTTGT
**BEST1**	Forward	AACTGAGCCTACCACACAACA
	Reverse	CGGATTCGACCTCCAAGCC
**COLA1**	Forward	TCACCAGGACAGAAGGGAAG
	Reverse	CTCTGGCACCTTTTGCTAGG
**COL4A4**	Forward	CTGGATTTGGAGAACGTACCG
	Reverse	TGATCCAATTCTGCCTGCAC
**FN1**	Forward	ATGATGAGGTGCACGTGTGT
	Reverse	CTCTTCATGACGCTTGTGGA
**PDGFRB**	Forward	AGCACCTTCGTTCTGACCTG
	Reverse	TATTCTCCCGTGTCTAGCCCA
**PDK4**	Forward	GGAGCATTTCTCGCGCTACA
	Reverse	ACAGGCAATTCTTGTCGCAAA
**PECAM1**	Forward	AACAGTGTTGACATGAAGAGCC
	Reverse	TGTAAAACAGCACGTCATCCTT
**PPARD**	Forward	CCAACAGATGAAGACAGATGCA
	Reverse	CTGAACGCAGATGGACCTCTA
**RPE65**	Forward	CCTGCTGGTGGTTACAAGAAA
	Reverse	CCTGCCTGTTACATGAGCTGT
**RXRA**	Forward	GAGCCCAAGACCGAGACCTA
	Reverse	AGCTGTTTGTCGGCTGCTT
**RXRB**	Forward	AGCCCCCAGATTAACTCAACA
	Reverse	GATTGCACATAGCCGTTTGC
**SERPINF1**	Forward	TTCAAAGTCCCCGTGAACAAG
	Reverse	GAGAGCCCGGTGAATGATGG
**TGFB1**	Forward	CAATTCCTGGCGATACCTCAG
	Reverse	AGATAACCACTCTGGCGAGTC
**VEGFA**	Forward	AGGGCAGAATCATCACGAAGT
	Reverse	AGGGTCTCGATTGGATGGCA
**VTN**	Forward	CACTATGCCGGAGGATGAGT
	Reverse	TCAGGATTCCCTTTGGACTG

Table S4. List of drugs, concentrations, and sources.

Antibody/Stain	Source	Dilution
Apolipoprotein E (ApoE)	Millipore	1:1000
Collagen IV (COL4)	Millipore	1:30
CRALBP	Gift from John C Saari and Thermo Fisher	1:2500
F4/80	AbD Serotec	1:500
Fibronectin (FN1)	Abcam	1:100
Iba1 (Ionized calcium-binding adapter molecule 1)	WAKO	1:200
PKCα	Abcam	1:200
Red-Green Opsin	Millipore	1:1000
Rhodopsin	Abcam	1:1000
SV2	Hybridoma bank	1:200
Phalloidin Tetramethylrhodamine B isothiocyanate	Sigma	1:500
